# High-Performance Nitric Oxide Gas Sensors Based on an Ultrathin Nanoporous Poly(3-hexylthiophene) Film

**DOI:** 10.3390/bios13010132

**Published:** 2023-01-13

**Authors:** Ganghoon Jeong, Seo Young Shin, Proscovia Kyokunzire, Hyeong Jun Cheon, Eunsol Wi, Minhong Woo, Mincheol Chang

**Affiliations:** 1 Graduate School, Department of Polymer Engineering, Chonnam National University, Gwangju 61186, Republic of Korea; 2 Alan G. MacDiarmid Energy Research Institute, Chonnam National University, Gwangju 61186, Republic of Korea

**Keywords:** nitric oxide, organic field-effect transistor, gas sensor, poly(3-hexylthiophene), nanoporous conjugated polymer film

## Abstract

Conjugated polymer (CP)-based organic field-effect transistors (OFETs) have been considered a potential sensor platform for detecting gas molecules because they can amplify sensing signals by controlling the gate voltage. However, these sensors exhibit significantly poorer oxidizing gas sensing performance than their inorganic counterparts. This paper presents a high-performance nitric oxide (NO) OFET sensor consisting of a poly(3-hexylthiophene) (P3HT) film with an ultrathin nanoporous structure. The ultrathin nonporous structure of the P3HT film was created via deposition through the shear-coating-assisted phase separation of polymer blends and selective solvent etching. The ultrathin nonporous structure of the P3HT film enhanced NO gas diffusion, adsorption, and desorption, resulting in the ultrathin nanoporous P3HT-film-based OFET gas sensor exhibiting significantly better sensing performance than pristine P3HT-film-based OFET sensors. Additionally, upon exposure to 10 ppm NO at room temperature, the nanoporous P3HT-film-based OFET gas sensor exhibited significantly better sensing performance (i.e., responsivity ≈ 42%, sensitivity ≈ 4.7% ppm^−1^, limit of detection ≈ 0.5 ppm, and response/recovery times ≈ 6.6/8.0 min) than the pristine P3HT-film-based OFET sensors.

## 1. Introduction

Nitrogen oxides (NO_x_) are generated as by-products of the combustion of nitrogen-containing fossil fuels, and they generally refer to compounds such as nitric dioxide (NO_2_), nitrous oxide (N_2_O), and nitric oxide (NO) [1,2]. In 1990, NO_x_ was classified as one of the criterion pollutants under the Clean Air Act regulations, because it is a hazardous gas that can cause serious damage to human health and the environment [1,3]. Many studies have been conducted to develop gas sensors that can detect and monitor NO_x_. However, most studies have focused on the detection of NO_2_ gas because NO_2_ molecules have a stronger electron affinity than other NO_x_ molecules (N_2_O and NO_2_) and can thus be more easily detected using an electrochemical gas sensor [1,4,5]. NO gas is one of the major components of NO_x_, which is generally known to cause acid rain, photochemical smog, and ozone depletion. Additionally, NO rapidly oxidizes to NO_2_, resulting in adverse effects on human health and the environment [6,7,8,9]. For example, NO gas substantially causes cyanosis, asthma, itching, diabetes, pulmonary inflammation, and fatality [10,11,12]. Hence, NO detection has become an important concern in industrial processing and pollution control.

A typical example of a commercial gas sensor is a metal oxide gas sensor that operates on the principle of resistance change due to analyte–active layer reduction/oxidation (redox) reactions at high temperatures [13,14]. However, metal oxide gas sensors have the disadvantage of requiring additional energy to achieve high gas sensing performance [15,16]. In this regard, conjugated polymer (CP)-based organic field-effect transistor (OFET) sensors are considerably interesting because of their various advantages, such as low cost, low power, and room temperature operation [1,17,18]. When CP-based OFET sensors are exposed to a target gas environment, their active layer undergoes changes in its density of states based on the redox properties of the gas molecules, thereby modulating the OFET channel current [1]. This sensing mechanism of metal oxide sensors is typically based on direct redox reactions (e.g., the Brønsted redox reaction), which require a high-temperature reaction environment. Conversely, OFET gas sensors can detect gas molecules at low power levels and room temperature via interactions such as Van der Waals forces, dipole–dipole interactions, and dipole-induced dipole interactions [1,19,20]. However, OFET-based sensors exhibit relatively low gas sensing performance because their sensing mechanism results in a weaker sensing signal than that generated by the sensing mechanism of direct redox [1].

Recently, several studies have been conducted to enhance the sensing properties of OFET-based sensors by optimizing the active layer morphology to increase interactions between the analyte and active layer and by minimizing the active layer thickness to improve the diffusion of analytes into the layer of the materials [21,22,23]. For example, some methods, such as colloidal templating [24], the breath figure method [1,25], and lithography [26], have been developed to fabricate porous CP films. However, these methods have limitations because of their complexity, high cost, and scale-up problems. Additionally, they generate nonuniform CP films with microporous structures [27,28].

Herein, we report a high-performance NO OFET sensor consisting of a poly(3-hexylthiophene) (P3HT) film with an ultrathin nanoporous structure that was fabricated via the shear-assisted phase separation (SAPS) of polymer blends, followed by selective solvent etching. In this study, P3HT was chosen as the CP model because of its good solubility in organic solvents and acceptable charge carrier mobility [21,29,30]. P3HT served as the active layer that recognizes NO molecules and transports its detection signal. Importantly, the ultrathin nanoporous structure of the P3HT film facilitated enhanced adsorption/desorption and diffusion of NO molecules, resulting in highly improved sensing performance. In particular, the nanoporous P3HT (N-P3HT) active-layer-based OFET NO sensor exhibited better sensing properties (i.e., responsivity ≈ 42%, sensitivity ≈ 4.7% ppm^−1^, limit of detection ≈ 0.5 ppm, and response/recovery times ≈ 6.6/8.0 min) than pristine P3HT (P-P3HT)-based OFET sensors.

## 2. Materials and Methods

### 2.1. Chemicals

Regioregular P3HT (regioregularity ≈ 96%; MW ≈ 69 kDa) was obtained from Rieke Metals Inc. Polystyrene (PS) (MW ≈ 192 kDa), chloroform (anhydrous grade), and 1,4-dioxane (anhydrous, 99.8%) were purchased from Sigma-Aldrich (St. Louis, MO, USA). All the materials were used without further purification.

### 2.2. CP-Based OFET Gas Sensor Fabrication

**Figure 1:**
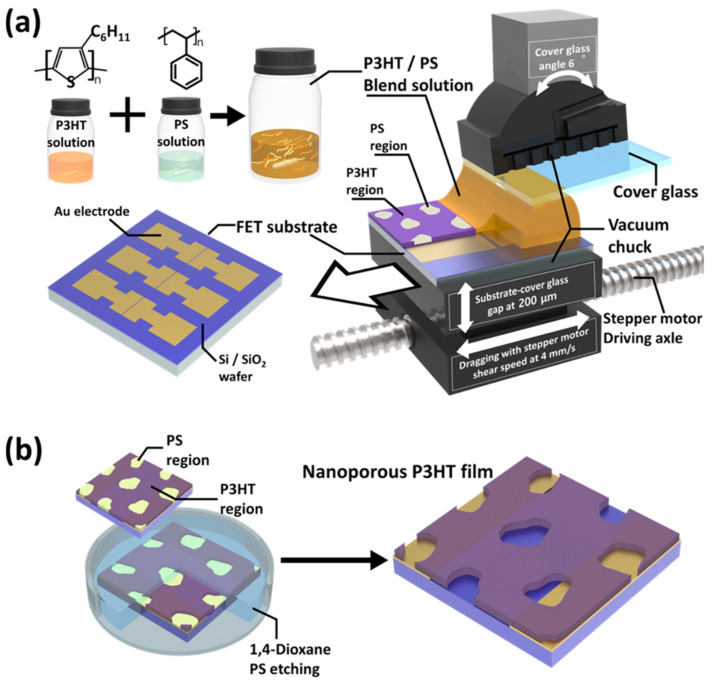
Schematic illustration of (**a**) the preparation of the poly(3-hexylthiophene) (P3HT)/polystyrene (PS) blend film (weight ratio = 70:30) and (**b**) the creation of the nanoporous P3HT (N-P3HT) film using the shear-assisted phase separation (SAPS) method and selective solvent etching.

The FET structure was constructed on a highly doped Si/SiO_2_ wafer substrate. Source and drain Au/Cr electrodes were photolithographically defined to have a channel length of 50 µm and a width of 2 mm and were deposited on this Si/SiO_2_ wafer substrate using a thermal evaporator (JVMS-23M151S, the Energy Convergence Core Facility, Chonnam National University, Republic of Korea). The deposited Au electrodes consisted of 50 nm contacts on a 3 nm Cr adhesion layer. A previously reported procedure was used to deposit the CP active layers by shear coating (4 mm s^−1^ of shearing speed) the polymer blend solution onto a precleaned FET substrate under ambient conditions [21]. For the shear coating, the FET substrate and a cover glass were placed on each vacuum chuck attached to a software-controlled microstage (Zaber X-LHM200A-E03), as shown in Figure 1a. The cover glass was positioned above the FET substrate and tilted at an angle of 6° relative to it, and the gap between them was fixed at 200 μm. The gap between the cover glass and the FET substrate was filled with approximately 20 μL of a P3HT/PS blend solution using a pipette. To prepare this P3HT/PS blend solution, P3HT and PS were each dissolved in chloroform at a concentration of 5 mg mL^−1^ and stirred with a magnetic stirrer at ~55 °C for at least 30 min. Thereafter, the P3HT and PS solutions were cooled to room temperature (22 °C) and then combined using a P3HT:PS mass ratio of 7:3 [21]. Subsequently, the P3HT/PS film was deposited on the FET substrate by utilizing a stepper motor to drag the substrate while the cover glass remained fixed on the chuck. The prepared OFET devices were annealed in a vacuum oven at 55 °C for over 12 h to remove any residual solvent in the deposited polymer film.

The phase-separated PS regions were removed from the P3HT/PS film that was deposited on the OFET substrate to generate a porous structure in the film (Figure 1b). The as-prepared OFET devices were immersed in a sufficient amount of 1,4-dioxane for more than 12 h. Subsequently, the OFET devices were removed from the 1,4-dioxane and dried with compressed air for a few seconds. The dried OFET devices were stored in a vacuum chamber for at least 12 h to remove any residual solvent.

### 2.3. Gas Sensing Evaluation and Characterization of the OFET Devices

A semiconductor analyzer (Keithley 4200-SCS) was used to measure the electrical properties of the OFET devices in an argon-filled glove box at room temperature. The charge carrier mobility was calculated from the saturation region (drain–source voltage (*V_DS_*) = −80 V) as follows:

(1) $$I_{DS}=\mu \left(C_{i}\frac{W}{2L}\right){\left(V_{GS}-V_{th}\right)}^{2},$$

where *I_DS_* is the drain–source current, μ is the charge carrier mobility, *C_i_* is the capacitance per unit area of the dielectric layer of the OFET device, *W* and L are the channel width and length, respectively, and *V_GS_* and *V_th_* are the gate–source and threshold voltages, respectively [31].

The NO sensor was tested using a homemade sensing chamber (volume: ~5 cm^3^) equipped with volumetric flow controllers and a data acquisition system. A certain concentration of NO gas and synthetic dry air (80% N_2_ and 20% O_2_) was introduced into the chamber through a volumetric flowmeter at a flow rate of 100 mL min^−1^. Standard NO gas (1000 ppm in N_2_, blending tolerance ±2%, Rigas Co. Ltd., Daejeon, Republic of Korea) was diluted with synthetic dry air to generate a gas mixture with various concentrations. The semiconductor analyzer was used as the data acquisition system to record real-time variations in the *I_DS_* value of the OFET sensor (*V_GS_* = 20 V and *V_DS_* = −80 V) at 3 min intervals. The responsivity of the sensor was calculated as follows:

(2) $$R\;\left(\%\right)=\frac{I_{\;NO}-I_{air}}{I_{air}}\times 100,$$

where *R* (%) is the responsivity, and *I_NO_* and *I_air_* represent the initial currents of the sensor under NO gas and synthetic air conditions, respectively. The sensitivity of the gas sensor is defined as S = R/C_NO_ (% ppm^−1^), where C_NO_ is the NO concentration (ppm) [21]. The response time is the time required for the responsivity to increase to 90%, while the recovery time is the time required for the responsivity to decrease to 10% from its maximum value upon exposure to NO and synthetic air at 10 min intervals [21]. The film thickness and pore size of the active layer of the OFET sensor were observed using atomic force microscopy (AFM; NX20, Park Systems, Suwon, Republic of Korea).

## 3. Results and Discussion

**Figure 2:**
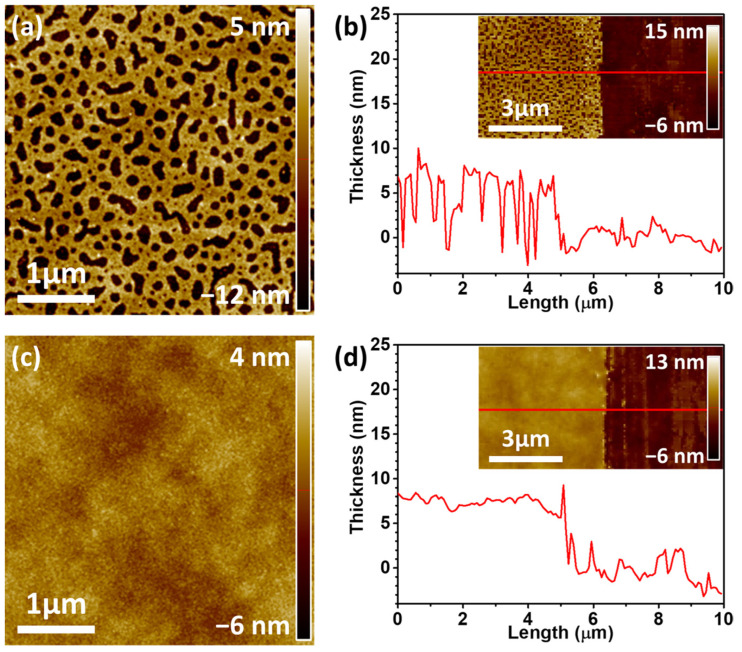
AFM topology images and corresponding film thickness profiles (measured along the red line) of the (**a**,**b**) N-P3HT film and (**c**,**d**) pristine P3HT (P-P3HT) film.

Generally, the phase separation of P3HT and PS occurs owing to their low miscibility caused by their different solubility parameters (δ_P3HT_ = 14.8 ± 0.2 MPa^1/2^ and δ_PS_ = 17.9 ± 0.2 MPa^1/2^) [32]. To prepare the N-P3HT films, the P3HT/PS weight ratio of the blend solution was fixed at P3HT:PS = 7:3, and the shear speed was fixed at 4 mm s^−1^ during the SAPS process, according to a previous study [21]. Generally, the film thickness changes as the shearing speed changes during deposition using solution shear coating. Three coating regimes are basically defined based on the shear coating speed: the evaporation regime, transition regime, and Landau–Levich regime [21,33]. Polymer films with large thicknesses and pores are created both in the evaporation regime (ca. 0.5–2.0 mm s^−1^) and Landau–Levich regime (ca. 8.0–40 mm s^−1^), owing to their slow and fast shear speeds, respectively [21]. Thus, an intermediate shear speed (i.e., 4.0 mm s^−1^) was selected from the shear speed range belonging to the transition regime to fabricate P3HT films with the smallest possible thickness and nanopores. After the blend films were deposited on the FET substrate using the SAPS method, the PS regions of the P3HT/PS blend films were removed via selective solvent etching using 1,4-dioxane, which was used because it acts as a bad solvent for P3HT but a good one for PS [34,35]. The resulting N-P3HT film had a film thickness and pore size of approximately 8.1 nm and 0.017 µm^2^, respectively (Figure 2a,b; Table 1). The P-P3HT film had a film thickness of approximately 7.8 nm, indicating that both films had similar thicknesses. The N-P3HT film exhibited a higher surface area ratio (defined as the surface area/geometric area) of 1.15% than the P-P3HT film (0.06%), owing to their pores (Table 1). These results indicate that the N-P3HT film has a larger surface area than the P-P3HT film [21].

**Figure 3:**
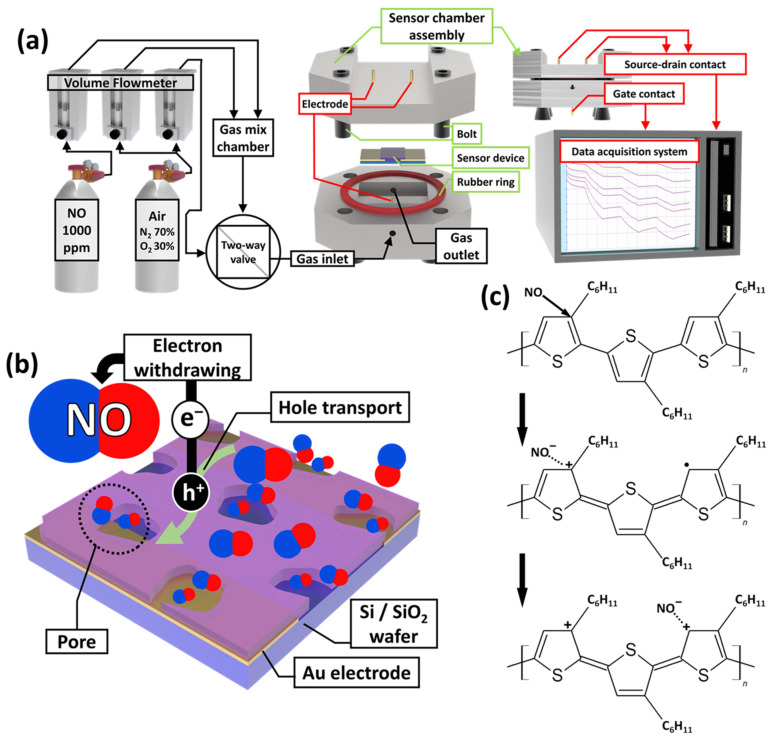
Schematic illustration of (**a**) the NO gas flow, gas chamber, and data acquisition system. (**b**) Gas sensing mechanism of the N-P3HT-based OFET NO gas sensor for detecting NO molecules. (**c**) P3HT doping mechanism by adsorption of NO molecules.

The N-P3HT OFET sensor was tested using a homemade sensing chamber connected to volumetric controllers and a data acquisition system to evaluate its electrical properties and sensing performance (Figure 3a). A standard NO gas concentration (1000 ppm) was diluted with synthetic dry air using a volume flowmeter to create gas/air mixtures with different NO concentrations (0.5, 1, 2, 3, 4, 5, 10, 15, 20, and 30 ppm). A NO gas/air mixture containing a certain NO concentration and synthetic dry air (i.e., set as 0 ppm of the target gas) was selectively injected into the homemade sensing chamber through a two-way valve. Note that the oxidation of NO gas was not considered in this study, as it was mixed and injected into the sensing chamber within seconds [36]. As the NO gas was injected into the gas chamber, the NO molecules interacted with the P3HT active layer of the OFET gas sensor. Consequently, the channel current of the P3HT OFET gas sensor increased owing to the electron-withdrawing properties of the NO molecules [37], which is similar to the hole-doping mechanism of p-type semiconductors (Figure 3b,c) [1,38,39].

**Figure 4:**
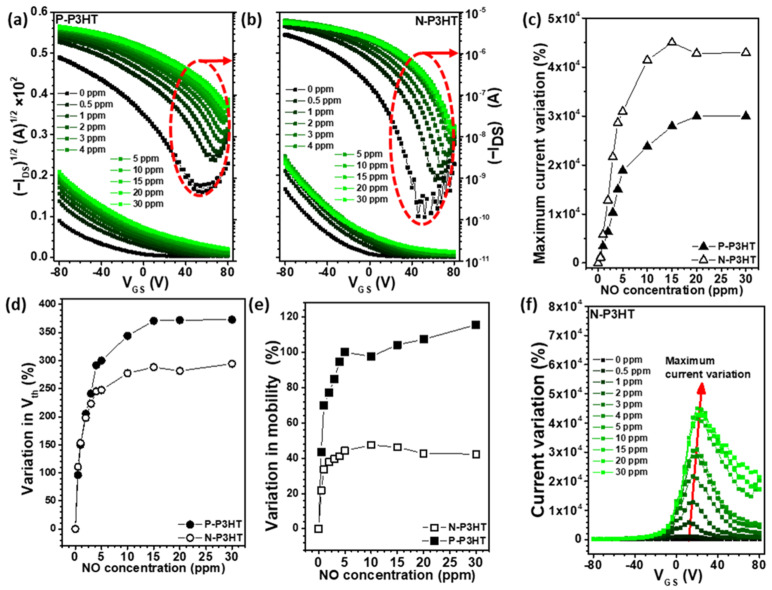
Transfer curves of the OFETs based on the (**a**) P-P3HT and (**b**) N-P3HT active layers. The variation in the (**c**) maximum current, (**d**) threshold voltage, and (**e**) charge carrier mobility of the N-P3HT OFET and P-P3HT OFET sensors upon exposure to various NO concentrations. (**f**) Current variation ((I_ON_ − I_0_)/I_0_, %) over the gate voltage of the N-P3HT film.

As shown in Figure 4a,b, a semiconductor analyzer system was used to record the real-time variation in the electrical properties as the NO concentration changed. Generally, gas analytes are detected based on variations in the output current, threshold voltage, or charge carrier mobility of OFET sensors [21,40,41]. When the P-P3HT and N-P3HT OFET sensors were exposed to 30 ppm NO gas, their maximum current variation (29,960% and 42,975%, respectively) became more prominent than their threshold voltage variation (373% and 294%, respectively) and charge carrier mobility variation (116% and 42%, respectively) (Figure 4c–e and Figure S1). It should be noted that the maximum current variation value calculated based on the initial current (I_0_) recorded by the first exposure of synthetic air differs from the responsivity calculated based on the current (I_air_) obtained by consecutive synthetic air exposure. This result indicates that measuring the output current variation of OFET sensors is more effective for detecting NO gas molecules than measuring their threshold voltage and charge carrier mobility. Additionally, selecting the optimum gate voltage of OFET sensors is crucial for amplifying their detection signal. Therefore, the I–V curves of the P-P3HT OFET and N-P3HT OFET sensors were obtained over various NO concentrations, as shown in Figure 4f and Figure S1a. The optimum gate voltage for both OFET sensors ranged between 10 and 30 V. Based on this result, a gate voltage of 20 V was selected and fixed for the NO sensing test over various NO concentrations. It should be noted that the N-P3HT OFET sensors had a higher charge carrier mobility than the P-P3HT OFET sensors (Figure 4e and Table S1). The high mobility of the N-P3HT OFET sensors is believed to be due to the PS chains that existed in the P3HT/PS blend films before PS removal. PS chains are known to facilitate the self-assembly of P3HT chains during the formation of P3HT/PS blend films (Figure S2), thereby improving the charge mobility of the blend films [42,43,44].

**Figure 5:**
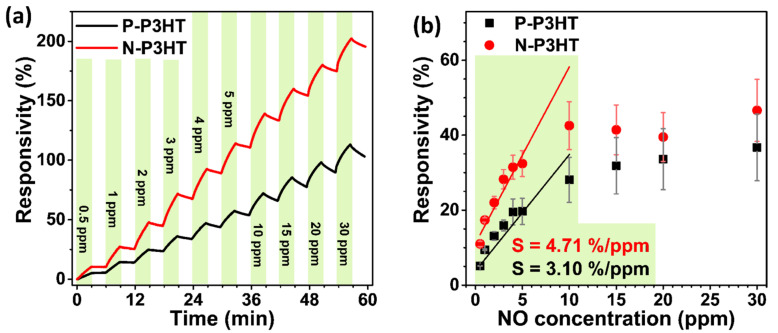
(**a**) Real-time responsivity and (**b**) responsivity vs. NO concentration plots of the P-P3HT and N-P3HT OFET sensors.

Figure 5 shows the real-time responsivity and sensitivity of the P-P3HT and N-P3HT OFET sensors to various NO concentrations. Both sensors clearly exhibited baseline shifts (Figure 5a) and responsivity saturation (Figure 5b) as the NO concentration increased. The baseline shift and sensitivity saturation are related to the incomplete desorption of NO from the P3HT active layer of the sensor devices [21,45,46]. Consequently, the N-P3HT OFET sensor had a stronger response to NO concentrations than the P-P3HT OFET sensor (Figure 5a,b; Table 2). Specifically, the responsivity of the P-P3HT and N-P3HT OFET sensors increased from 5.1% to 28.1% and 10.1% to 42.5%, respectively, as the NO concentration increased from 0 to 10 ppm. However, the responsivity was nearly saturated when the NO concentration was further increased to 30 ppm, implying that the active sites of the P3HT layer were almost occupied with NO molecules [21]. Similarly, the sensitivity was calculated from the slope of the responsivity—NO concentration plots within a range of 0.5–10 ppm of NO [20,21], and the N-P3HT OFET sensor was found to have a higher sensitivity (4.71% ppm^−1^) than the P-P3HT OFET sensor (3.10% ppm^−1^) (Figure 5b). The excellent sensing behavior of the N-P3HT OFET sensor is attributed to its relatively large surface area (active site) and fast analyte diffusion caused by the nanoporous structure of the N-P3HT films. It should be noted that the responsivity of the P-P3HT and N-P3HT OFET sensors was not recorded at NO gas concentrations below 0.5, indicating that the limit of detection of both sensors was ~0.5 ppm.

**Figure 6:**
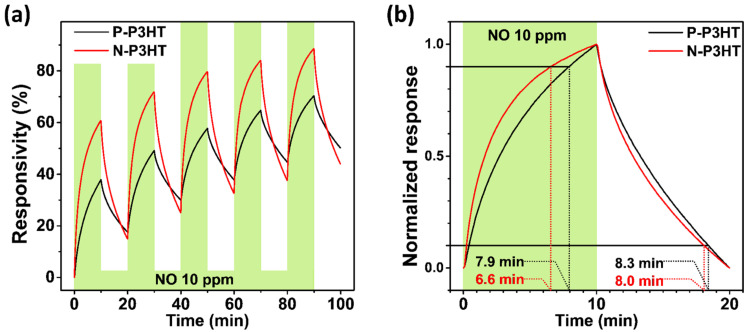
(**a**) Time-dependent and (**b**) normalized responsivities of the P-P3HT OFET and N-P3HT OFET sensors.

The reproducibility, response time, and recovery time of the N-P3HT OFET sensors, which are important parameters for practical application, were investigated by introducing 10 ppm NO gas and bare synthetic dry air alternately with a 10 min interval for 5 cycles, as shown in Figure 6 and Table S2. It should be noted that in general, the sensor signal of P3HT-based OFET sensors is not fully recovered due to the partial desorption of target gas molecules from the active layer of P3HT-based sensors, which also causes a corresponding baseline drift, as shown in Figure 6a [45,46,47,48,49]. As shown in Figure 6a, the N-P3HT OFET sensors exhibited a more stable response with a higher responsivity than the P-P3HT OFET sensors over the repeated cycles, demonstrating their good reproducibility and thereby practicability as gas sensors. Moreover, the N-P3HT OFET sensor exhibited better response and recovery times (6.6 and 8.0 min, respectively) than the P-P3HT OFET sensor (7.9 and 8.3 min, respectively) (Figure 6b and Table S2). The N-P3HT OFET sensor exhibited better sensing performance than the P-P3HT OFET sensor because of its superior morphological feature (i.e., its nanoporous structure), which can provide abundant active sites and facilitate rapid analyte adsorption, desorption, and diffusion [21]. Note that the sensing performance (i.e., responsivity, response/recovery time, and LOD) of the N-P3HT OFET sensors is comparable or superior to that of previously reported other types of gas sensors (e.g., inorganic and organic resistor type sensor) operated at room temperature (Table S3) [50,51,52,53]. Moreover, the sensing performance of the N-P3HT OFET sensors is better due to the easy control of pore size and the realization of smaller pore sizes, compared to that in previously reported studies on CP-based OFET NO sensors with a similar structure [1].

## 4. Conclusions

Herein, we report highly improved NO gas sensing using an OFET sensor based on an ultrathin N-P3HT film. The resulting N-P3HT OFET sensor exhibited better NO gas sensing performance (responsivity ≈ ~42%, sensitivity ≈ ~4.7% ppm^−1^, response time ≈ 6.6 min, and recovery time ≈ 8.0 min) than the P-P3HT OFET sensor (28%, 3.1% ppm^−1^, 7.9min, and 8.3 min, respectively) at room temperature. This remarkable improvement was attributed to the nanoporous structure of the N-P3HT film, which (1) provided numerous active sites for NO gas molecules to interact with charge carriers in the P3HT film, resulting in improved responsivity, and (2) enabled rapid analyte diffusion, adsorption, and desorption, thereby improving the response and recovery times. Moreover, the N-P3HT OFET sensor showed excellent reproducibility with a stable response for five sensing cycles, demonstrating that it can be used for gas sensing. The N-P3HT OFET sensor presented herein could be a promising platform for detecting and monitoring gases that are harmful to human health and the environment.

## Figures and Tables

**Table 1:** Morphological properties of the P-P3HT and N-P3HT films prepared using the SAPS method at a shear speed of 4 mm s^−1^ and a solute/solvent ratio of 5 mg/mL.

	P-P3HT	N-P3HT
Film thickness (nm)	7.8 ± 0.5	8.1 ± 0.3
Pore size (µm^2^)	-	(1.7 ± 0.07) × 10^−2^
Surface area ratio (%)	0.061 ± 0.001	1.150 ± 0.026

**Table 2:** Responsivity (%) of the P-P3HT-based and N-P3HT-based OFET sensors to various NO gas concentrations (on/off intervals of 3 min; *V_DS_* = −80 V and *V_GS_* = 20 V).

NO Concentration (ppm)	Responsivity (%)	
	P-P3HT	N-P3HT
0.5	5.10 ± 0.01	10.1 ± 0.01
1	9.44 ± 0.01	17.4 ± 0.01
2	13.1 ± 0.01	22.0 ± 0.02
3	15.9 ± 0.01	28.2 ± 0.02
4	19.5 ± 0.01	31.4 ± 0.03
5	19.7 ± 0.03	32.4 ± 0.03
10	28.1 ± 0.06	42.5 ± 0.06
15	31.8 ± 0.07	41.4 ± 0.07
20	33.6 ± 0.08	39.5 ± 0.06
30	36.7 ± 0.09	46.6 ± 0.08

## Data Availability

The datasets generated and/or analyzed during this study are available upon reasonable request from the corresponding author.

## Supplementary Materials

The following supporting information can be downloaded at: https://www.mdpi.com/article/10.3390/bios13010132/s1, Figure S1: (a) Current variation ((I_ON_ − I_0_)/I_0_) over the gate voltage of the P-P3HT film. (b) Threshold voltage and (c) charge carrier mobility of the corresponding P-P3HT and N-P3HT OFETs based on variations in the NO concentration; Figure S2: Normalized ultraviolet–visible absorption spectra of the P-P3HT and N-P3HT films. The P-P3HT film was fabricated using the shear coating method, and the N-P3HT film was fabricated using the SAPS method. The shear speed was set at 4 mm s^−1^; Table S1: Electrical properties of the P-P3HT-based and N-P3HT-based OFET devices under various NO concentrations; Table S2: Responsivity (%) and response/recovery times (min) of the P-P3HT-based and N-P3HT-based OFET NO sensors in relation to a 10 ppm NO concentration for five cycles (on/off intervals of 10 min); Table S3: Comparison of the gas sensing characteristics of various nitric oxide gas sensors.
