# Supplementary material for: High-Performance Nitric Oxide Gas Sensors Based on an Ultrathin Nanoporous Poly(3-hexylthiophene) Film
Source: Biosensors (Basel). 2023 Jan 13;13(1):132. doi: 10.3390/bios13010132 (PMC9856169; doi:10.3390/bios13010132)
Supplement: Supplementary file 1 [file biosensors-13-00132-s001.zip › biosensors-2103996-supplementary.pdf]

biosensors

MDPI

Supplementary Materials

High-performance nitric oxide gas sensors based on an ultrathin nanoporous poly(3-hexylthiophene) film

Ganghoon Jeong [1], Seo Young Shin [1], Proscovia Kyokunzire [1], Hyeong Jun Cheon [1], Eunsol Wi [1], Minhong Woo [1] and Mincheol Chang [1,2,*]

[1] Graduate School, Department of Polymer Engineering, Chonnam National University, Gwangju 61186, South Korea
[2] Alan G. MacDiarmid Energy Research Institute, Chonnam National University, Gwangju 61186, South Korea
* Correspondence: mchang35@chonnam.ac.kr

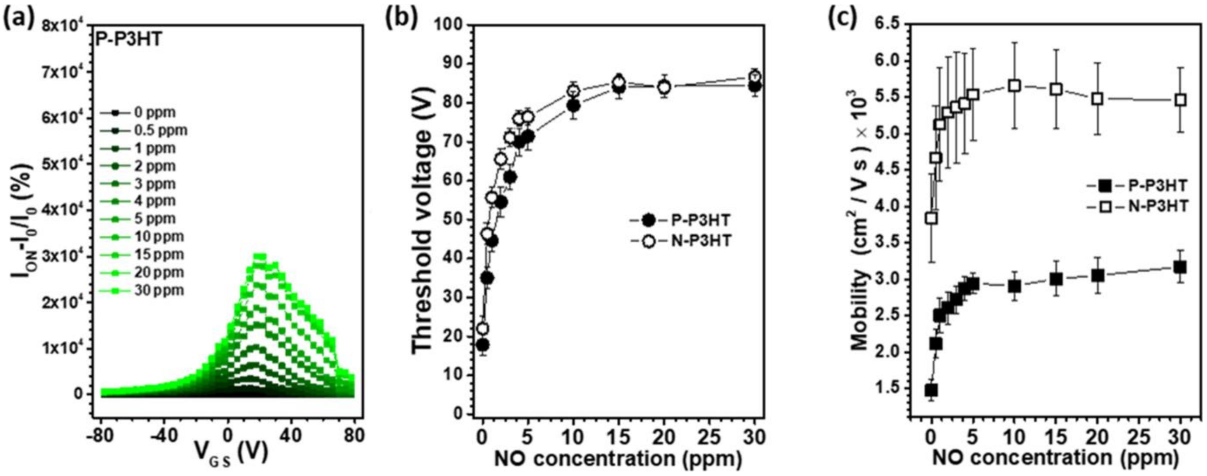

Figure S1. (a) Current change ratio (($I_{ON} - I_0$) / I_0) over the gate voltage of the P-P3HT film. (b) Threshold voltage evolution of the corresponding P-P3HT-based and N-P3HT-based OFETs based on variations in the NO concentration. (c) Charge carrier mobility of the corresponding OFETs upon exposure to various NO gas concentrations.

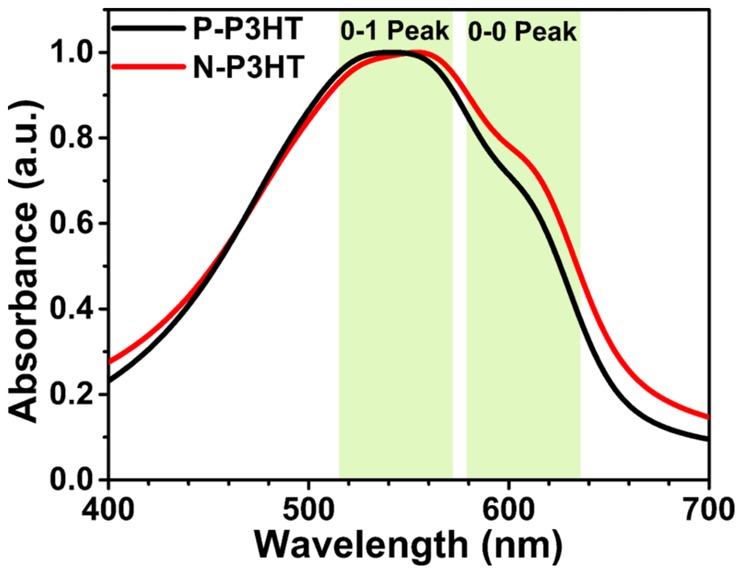

Figure S2. Normalized UV-vis absorption spectra of the P-P3HT and N-P3HT films. The P-P3HT film was fabricated using the shear coating method, and the N-P3HT film was fabricated using the SAPS method. The shear speed was set at 4 mm s^{-1}.

Table S1. Electrical properties of the P-P3HT-based and N-P3HT-based OFET devices under various NO concentrations.

NO concentration (ppm)		P-P3HT	N-P3HT
**Threshold voltage (V)**	0	18 ± 2.7	22 ± 3.1
	0.5	35 ± 2.6	46 ± 2.8
	1	44 ± 2.8	55 ± 2.6
	2	54 ± 3.7	65 ± 2.5
	3	60 ± 3.3	71 ± 2.2
	4	69 ± 3.5	75 ± 2.0
	5	71 ± 3.6	76 ± 2.3
	10	79 ± 3.4	83 ± 2.2
	15	84 ± 2.9	85 ± 2.0
	20	84 ± 3.0	83 ± 1.7
	30	84 ± 2.7	86 ± 2.0
**Charge carrier mobility ($cm^2\ V^{-1}\ s^{-1}$)**	0	$(1.47 \pm 0.15) \times 10^{-3}$	$(3.84 \pm 0.61) \times 10^{-3}$
	0.5	$(2.11 \pm 0.20) \times 10^{-3}$	$(4.67 \pm 0.72) \times 10^{-3}$
	1	$(2.50 \pm 0.23) \times 10^{-3}$	$(5.12 \pm 0.77) \times 10^{-3}$
	2	$(2.60 \pm 0.22) \times 10^{-3}$	$(5.29 \pm 0.76) \times 10^{-3}$
	3	$(2.72 \pm 0.18) \times 10^{-3}$	$(5.36 \pm 0.76) \times 10^{-3}$
	4	$(2.86 \pm 0.17) \times 10^{-3}$	$(5.42 \pm 0.69) \times 10^{-3}$
	5	$(2.94 \pm 0.14) \times 10^{-3}$	$(5.53 \pm 0.63) \times 10^{-3}$
	10	$(2.91 \pm 0.19) \times 10^{-3}$	$(5.66 \pm 0.60) \times 10^{-3}$
	15	$(3.00 \pm 0.24) \times 10^{-3}$	$(5.61 \pm 0.54) \times 10^{-3}$
	20	$(3.05 \pm 0.25) \times 10^{-3}$	$(5.48 \pm 0.49) \times 10^{-3}$
	30	$(3.17 \pm 0.22) \times 10^{-3}$	$(5.46 \pm 0.45) \times 10^{-3}$

Table S2. Responsivity (%) and response/recovery times (min) of the P-P3HT-based and N-P3HT-based OFET NO sensors in relation to a 10 ppm NO gas concentration for repeated cycles (on/off intervals of 10 min; $V_{DS} = -80$ V and $V_{GS} = 20$ V).

Cycle number		P-P3HT	N-P3HT
**Responsivity (%)**	1	37.9	60.7
	2	31.6	56.8
	3	27.8	54.5
	4	26.9	51.4
	5	25.7	51.0
Response time (min)	1	7.95	6.87
	2	8.03	6.68
	3	8.03	6.60
	4	7.93	6.60
	5	7.93	6.60
Recovery time (min)	1	8.12	7.95
	2	8.30	7.95
	3	8.38	7.95
	4	8.38	8.03
	5	8.30	8.12

Table S3. Comparison of the gas sensing characteristics of various NO gas sensors.

**Material Structure**	**NO [ppm]**	**R [%]**	**S [% ppm^{-1}]**	**LOD [ppm]**	t_{res} **[min]**	t_{rec} **[min]**
N-P3HT OFET (This work)	10	54.9 ± 1.6	4.71	~0.5	6.67 ± 0.05	8.00 ± 0.03
TiO_2 / TSV structure Resistor [1]	4	16.7	~3	-	1.82	2.40
SnO_2 / Ni- NCG Resistor [2]	40	15	~1	-	15.0	18.3
PEDOT-PSS : TiO_2 Resistor [3]	250	96	~0.3	1	3.87	0.92
PCDTBT Resistor [4]	100	80.6	-	5	5.0	35.0

Reference

1. Yeh, Y.-M.; Chang, S.-J.; Wang, P.-H.; Hsueh, T.-J. A TSV-Structured Room Temperature p-Type TiO_2 Nitric Oxide Gas Sensor. *Applied Sciences* **2022**, *12*, 9946.
2. Gupta Chatterjee, S.; Dey, S.; Samanta, D.; Santra, S.; Chatterjee, S.; Guha, P.; Chakraborty, A.K. Near room temperature sensing of nitric oxide using SnO_2/Ni-decorated natural cellulosic graphene nanohybrid film. *Journal of Materials Science: Materials in Electronics* **2018**, *29*, 20162-20171.
3. Khasim, S.; Pasha, A.; Badi, N.; Imran, M.; Al-Ghamdi, S. Development of high-performance flexible and stretchable sensor based on secondary doped PEDOT–PSS: TiO_2 nanocomposite for room-temperature detection of nitric oxide. *Journal of Materials Science: Materials in Electronics* **2021**, *32*, 7491-7508.
4. Gusain, A.; Joshi, N.J.; Varde, P.; Aswal, D. Flexible NO gas sensor based on conducting polymer poly [N-9′-heptadecanyl-2, 7-carbazole-alt-5, 5-(4′, 7′-di-2-thienyl-2′, 1′, 3′-benzothiadiazole)](PCDTBT). *Sens. Actuators B Chem.* **2017**, *239*, 734-745.
